# Molecular Mechanisms that Regulate Epidermal Growth Factor Receptor Inactivation

**DOI:** 10.4137/cmo.s498

**Published:** 2008-02-09

**Authors:** Brian P. Ceresa, Phillip A. Vanlandingham

**Affiliations:** Department of Cell Biology, University of Oklahoma Health Sciences Center, Oklahoma City, Oklahoma 73190

**Keywords:** EGFR, endocytosis, degradation, MVB

## Abstract

The Epidermal Growth Factor Receptor (EGFR) is the prototypical receptor tyrosine kinase (RTK). These cell surface receptors are integral membrane proteins that bind ligands on their extracellular domain and relay that information to within the cell. The activated EGFR regulates diverse cell fates such as growth, proliferation, differentiation, migration, and apoptosis. These signaling properties are important for the appropriate development and maintenance of an organism. However, when inappropriately controlled, due to EGFR overexpression or hyperactivation, these signaling events are characteristic of many cancers. It remains unclear whether the uncontrolled EGFR activity leads to cell transformation or is a consequence of cell transformation. Regardless of the cause, increased EGFR activity serves both as a biomarker in the diagnosis of some cancers and is a molecular target for anti-cancer therapies. The promising results with current anti-EGFR therapies suggest that the receptor is a viable molecular target for a limited number of applications. However, to become an effective therapeutic target for other cancers that have elevated levels of EGFR activity, current approaches for inhibiting EGFR signaling will need to be refined. Here we describe the molecular mechanisms that regulate EGFR inactivation and discuss their potential as therapeutic targets for inhibiting EGFR signaling.

## The ErbB Family

The EGFR, or ErbB-1, is one of four members of the ErbB family that also includes ErbB-2, ErbB-3 and ErbB-4 (Carpenter, 2003; Citri et al. 2003). The EGFR is expressed as a monomeric 1186 amino acid transmembrane protein with approximately half of the protein extracellular (621 amino acids) (Ullrich et al. 1984). The receptor contains three important regions—the extracellular ligand binding domain, a transmembrane-anchoring domain, and an intracellular intrinsic kinase domain (Chinkers and Brugge, 1984; Downward et al. 1984a). The other ErbB family members are structurally similar, but differ in their preference for ligands, ability to associate with other family members, tissue distribution, and signaling properties (Wiley, 2003).

**Figure 1:**
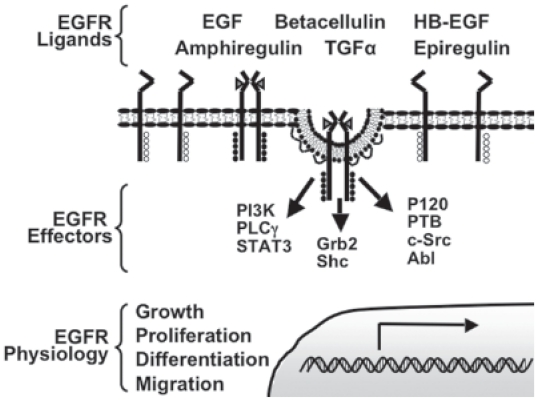
Schematic of EGFR activation Inactive EGFR exist as monomers on the plasma membrane. Upon binding of one of six endogenous ligands, two monomers dimerize and activate the receptor’s intrinsic kinase domain. The active kinase domain of one EGFR monomer transphosphorylates tyrosine residues on carboxyl terminus of its receptor pair. Once activated, the phosphotyrosines serve as docking site for downstream effectors, which include enzymes, adaptor proteins, and other regulatory molecules. Signaling from effectors integrates to modulate cell physiology, some of which are indicated. Phosphatidyl inositol 3-kinase (PI3K), phospholipase Cγ (PLCγ), signal transducers and activators of transcription 3 (STAT3), Growth factor receptor-bound protein 2 (Grb2), Src homology containing protein (Shc), p120 ras GTPase activating protein (P120), phosphatase B (PTB), cellular sarcoma (c-Src), and Abl.

Activation of the EGFR is dependent on binding ligand in the extracellular space that has been secreted in either an autocrine or paracrine manner (Fig. 1). There are six unique endogenous ligands for the EGFR helping generate diverse signals: EGF, transforming growth factor α (TGF-α) heparin-binding EGF (HB-EGF), amphiregulin, betacellulin, and epireglin (Harris et al. 2003). These ligands differ from one another in their regulated secretion, tissue distribution, and binding properties. Studies in which specific ligands have been ablated, either surgically or genetically, indicate that often one ligand can compensate for another (Tsutsumi et al. 1987). When three different ligands (EGF, TGF-α, and amphiregulin) were simultaneously knocked out of C57 black mice, there were defects in the skin, eye development, mammary gland development, and coat hair. When only one or two of the three ligands were ablated, there were only subtle phenotypes. One of which was mammary gland development when amphiregulin was knocked out in conjunction with TGFα or EGF (Luetteke et al. 1999). These findings suggest there are complementary roles for these ligands and underscore the difficulty in understanding receptor physiology in the context of a whole animal.

The EGFR is expressed in a variety of tissues (Yano et al. 2003). The EGFR is also critical to the development of tissues of epithelial, mesenchymal, and neuronal origin. EGFR knockout mice have been bred from different genetic backgrounds, showing either embryonic or postnatal lethality with multiple organ defects (Miettinen et al. 1995; Sibilia and Wagner, 1995). These findings point to the essential role of the EGF receptor in the development and tissue homeostasis of organisms in which it is expressed. Thus, the complete loss of EGFR function is deleterious to the organism.

## EGFR Activation

Ligand binding initiates receptor activation by inducing a conformational change and allowing for dimerization of two EGF receptor monomers (Ferguson et al. 2003). Dimerization leads to transphosphorylation of tyrosine residues on the cytoplasmic tail of one receptor by the intracellular kinase domain of the corresponding dimer (Lammers et al. 1990). Tyrosine phosphorylation is the essential activation step in EGFR signal transduction as these residues serve as docking sites for downstream signaling molecules containing Src homology 2 (SH2) or phosphotyrosine binding (PTB) domains. Signaling pathways activated by the EGFR include the phosholipase C gamma (PLCγ), mitogen-activated protein kinase (MAPK), and phosphatidylinositol 3 kinase (PI3K), Signal Transducers and Activators of Transcription 3 (STAT3) and Akt pathways (Yarden and Sliwkowski, 2001). The activities and biochemical changes induced by these signaling pathways integrate to mediate the specific modulation in cell biology such as cell growth, proliferation, differentiation, migration, and regulation of apoptosis (Jorissen et al. 2003).

## EGFR and Cancer

The first link between EGFR and malignant transformation came from studies that showed the EGFR is a homolog of the avian erythroblastosis virus v-erbB oncogene (Downward et al. 1984b). Several years later, a direct link between increased signaling from the EGFR and malignant growth was found in Epstein-Barr virus infected cells, a condition already known to lead to epithelial malignancies (Miller et al. 1995). Clinical studies provided supporting evidence that many human tumors and cell lines derived from human tumors overexpress EGFRs. Together, these data pointed to a potential role for the EGFR in tumor formation (Libermann et al. 1985; Merlino et al. 1984; Yamamoto et al. 1986). The hypothesized link between EGFR and cancer was strengthened when *in vitro* studies showed infection of NIH-3T3 cells with either retrovirus encoding EGFR, or a eukaryotic vector encoding EGFR cDNA, induced a transformed phenotype (Di Fiore et al. 1987; Velu et al. 1987).

EGFR overexpression and/or hyperactivation is associated with a number of cancers such as breast, ovary, renal, non-small cell lung, head and neck, colorectal, pancreatic, prostate, cervical and bladder (Sebastian et al. 2006). Overexpression is used as an indicator of poor prognosis in breast, ovarian, and head and neck cancers (Fischer-Colbrie et al. 1997; Ishitoya et al. 1989; Magne et al. 2001; Sebastian et al. 2006). In the clinic, increased EGFR expression has also been implicated with resistance to hormonal therapies in advanced stage breast cancers (Newby et al. 1997).

Although, it remains controversial as to whether EGFR overexpression is the cause of the cancer or a secondary consequence, the strong association between the EGFR and cancer has made it a natural candidate as an anti-cancer chemotherapeutic. Enhanced EGFR signaling can arise from a variety of mechanisms including receptor overexpression, mutations leading to constitutive activation, increased ligand production, or defective inactivation (Zandi et al. 2007). Early studies by Haigler and Carpenter indicated that in cultured cells, inhibition of ligand binding with an EGFR specific antibody could prevent DNA synthesis (Haigler and Carpenter, 1980). Since then, the EGFR has been a major molecular target in the treatment of cancer.

## Current EGFR-Targeted Inhibition Strategies

From the work in tissue culture, two strategies have emerged for inhibiting uncontrolled cell growth arising from EGFR overexpression or hyperactivation—monoclonal antibodies (MAbs) and tyrosine kinase inhibitors (TKIs). These approaches share the same goal of inhibition of receptor activity but differ in their molecular mechanism. MAbs target the extracellular portion of the receptor whereas the TKIs inhibit the intracellular portion. Multiple drugs from each class have been approved by the FDA for the treatment of certain types of cancers.

There are two monoclonal antibodies that have approval from the Food and Drug Administration (FDA)—Cetuximab (Erbitux) [February 2004] and Vectibix (Panitumumab) [September 2006]. These antibodies are used therapeutically for the treatment of metastatic colorectal cancer and cancers of the head and neck. Both Cetuximab and Vectibix inhibit binding of ligands to the EGFR causing a decrease in basal and ligand mediated receptor activation (Harari et al. 2007; Huang et al. 1999; Moroni et al. 2005). The decreased receptor activity inhibits cell growth, induces apoptosis, and decrease the production of other cellular factors associated with cancer progression and metastasis, such as matrix metalloproteinases, and vascular endothelial growth factor (Astsaturov et al. 2006; Zhu, 2007). Currently, the MAbs are used in combination with other agents or alone when the cancer is refractory to standard therapy.

The other class of EGFR inhibitors, TKIs, are small molecules that block EGFR activity by competing with ATP for use as a substrate by the receptor’s intrinsic kinase domain. The FDA has approved two EGFR-specifc TKIs, Iressa (Gefitinib) [December 2004] and Tarceva (Erlotinib) [November 2004], for the treatment of non-small cell lung carcinoma (Morgillo et al. 2007). A third FDA-approved drug, Lapatinib, can inhibit the kinase activity of both the EGFR and ErbB2. Lapatinib is used in combination with Herceptin for the treatment of HER2/neu positive breast cancers that are resistant to other therapies (Moy et al. 2007). Like the MAbs the mechanism of action for the TKIs is to block the activation of downstream signaling pathways. Interestingly, Gefitinib has been shown to be most effective in ~10% of non-small cell lung carcinoma patients with mutations around the ATP binding domain of the EGFR (Lynch et al. 2004).

Additional strategies targeting the EGFR focus on the rate at which new receptors are made and target the mRNA that encodes the EGFR. These approaches include EGFR-specific RNA interference (O’Grady et al. 2005), antisense oligonucleotides (Melisi et al. 2004), and ribozymes (Yamazaki et al. 1998). While these are effective approaches in tissue culture models, the methodology for delivering RNAi, oligonucleotides, and ribozymes to patients requires further development.

## Limitations of the Current Therapies that Target the EGFR

It should be noted that there are some restrictions to the FDA approved drugs that inhibit EGFR signaling and cancer growth. First, despite the wide range of cancers that are characterized by EGFR overexpression and/or hyperactivation, these drugs have only been approved for a limited number of cancers. However, both classes of drugs are currently enrolling patients in Phase 2 and Phase 3 trials for the treatment of other EGFR positive cancers, such as cervical, skin, myelogenous leukemia, prostate and glioblastomas (ClinicalTrials.gov, 2007).

Second, MAb and TKI therapies have numerous unpleasant side effects. Dermatological (rash, light sensitivity, and acne) and gastrointestinal (diarrhea, loss of appetite, and nausea) side effects have been reported for both therapeutic approaches (Rocha-Lima et al. 2007). Since proper EGFR function is required for normal skin and gastrointestinal mucosal regeneration (Grazul-Bilska et al. 2003; Jones et al. 1999), these toxicities are in all likelihood due to EGFR inhibition and aberrant EGFR-mediated regulation of tissue homeostasis.

## Novel Targets for Attenuating EGFR Signaling in Cancer

The treatment of patients with tumors that overexpress EGFRs could benefit from novel methods of inhibiting EGFR signaling. Alternative approaches to targeting the EGFR need to take into consideration the empirically determined strengths and weaknesses of the current therapies.

Rather than inhibit the activation of the EGFR, an alternative approach would be to accelerate the rate of receptor inactivation. Strategies to attenuate the activated EGFR in cancers by accelerating the endocytic process have been largely overlooked. Components of the endocytic pathway could be stimulated to accelerate the normal rate of signal attenuation. At this time, there are no agents under consideration, but that likely reflects the lack of molecular details regarding the EGFR signal termination and which molecules would be the best candidates.

Despite the incomplete understanding of this process, there are some obvious advantages to this strategy. By decreasing the duration of the active EGFR, the signaling necessary for cellular homeostasis would still be permitted while uncontrolled cell growth and replication would be inhibited. Second, those cells with the highest levels of receptor expression and/or activity would be affected the most. Third, by targeting receptor inactivation, there is no discrimination between uncontrolled cell growth that arises due to receptor overexpression versus receptor hyperactivation. This may allow such compounds have a broader applicability.

**Figure 2:**
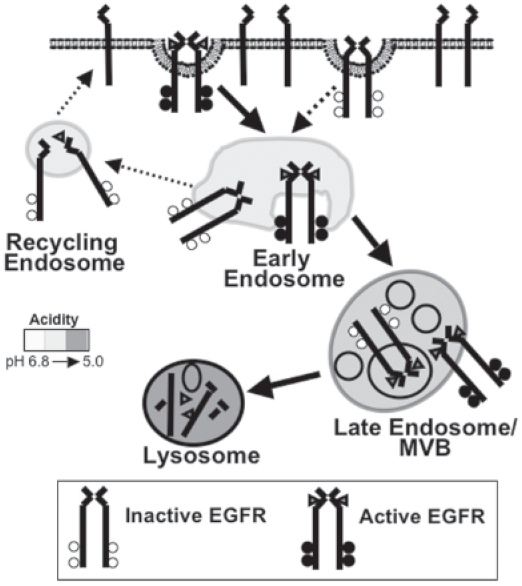
Ligand stimulated EGFR Endocytic trafficking (indicated by solid arrows) Ligand stimulation accelerates the rate of EGFR internalization via clathrin-coated pits. Following invagination and pinching off, the resulting clathrin-coated vesicle sheds its clathrin and delivers its cargo to the early endosome. In the early endosome, the cargo is sorted for delivery to its appropriate cellular location. In most cases, the EGFR is delivered to the lysosome for degradation. Early endosome matures into a late endosome/multiviesicular body (MVB). The contents of the late endosome/MVB are delivered to a lysosome for degradation. Indicated with dashed arrows are other possible routes of endocytic trafficking. A small percentage of total EGFR internalizes via clathrin-coated pits in a ligand independent manner (~1%–2%/min). In addition, unliganded EGFR will traffic from the early endosome to the plasma membrane via a recycling endosome upon ligand dissociation.

Below we discuss four potential mechanisms for signal termination of the activated EGFR from within the endocytic pathway: 1) dissociation of the ligand:receptor complex, 2) phosphatase-mediated receptor dephosphorylation 3) sequestration of the activated EGFR from effector molecules, and 4) lysosomal degradation of the receptor (Fig. 2). These molecular mechanisms have been shown to be effective ways of attenuating EGFR signaling in tissue culture models, but it remains unclear which ones are physiologically important. Nevertheless, at this point, they all remain as viable pharmacological targets. This discussion will include the pros and cons of each mechanism.

## EGFR Endocytic Trafficking

Ligand binding mediates two related functions. First, as mentioned above, is the initiation of downstream signaling pathways. Second, the ligand: receptor complex is internalized. This process, termed endocytosis, reduces the amount of receptor available for ligand binding on the cell surface as well as inactivates the receptor through dephosphorylation and/or receptor degradation. In addition, the internalization of the receptor physically moves the receptor through various endocytic compartments, and thereby changes the downstream effectors with which it has contact. Ligand-mediated receptor endocytosis has historically been overlooked as a molecular mechanism to attenuate the signaling of the EGFR.

There is a steady-state cycle in which the EGFR is slowly internalized (~1%–2% of the total receptor population/min) and rapidly recycled to the cell surface (Lund et al. 1990). However, ligand binding accelerates this process and induces a more dramatic redistribution of the receptor from the plasma membrane and directs the activated EGFR to the lysosome for degradation (Fig. 2). The EGFR is the only member of the ErbB family shown to undergo ligand-mediated internalization. In cells that express multiple ErbB family members, receptors that heterodimerize with the EGFR prevent endocytosis of the entire ligand:receptor complex (Baulida et al. 1996, Lenferink et al. 1998).

At the plasma membrane, ligand-bound EGFRs move laterally along the cell surface to a plasma membrane domain whose intracellular face is enriched with clathrin. The membrane domain invaginates to give rise to a clathrin-coated pit that eventually pinches off to form a clathrin-coated vesicle containing the ligand:receptor complex. Once inside the cell, the clathrin is shed from the vesicle and is now referred to as a primary endocytic vesicle. This vesicle fuses with the early endosome, and delivers the EGF:EGFR complex. Through endosomal maturation, the cargo arrives in a second endosomal compartment often referred to interchangeably as the multivesicular body (MVB) or late endosome. During this maturation, membrane structures form in the lumen, called intralumenal vesicles. In these internal vesicles, the EGFR is oriented such that the carboxyl terminal phosphotyrosines no longer have access to effector proteins. Finally, cargo is transferred to the lysosome by fusion of the endosome with lysosome, and degraded in the acidic, protease rich environment (Sorkin and von Zastrow, 2002).

Ligand-mediated endocytosis has always been recognized as a means of regulating EGFR signaling, but the mechanisms of regulation are still being discovered. Early studies by Wells et al. used truncation mutants of the EGFR that retained the ability to signal, but could not internalize. NIH 3T3 cells expressing these mutants underwent EGF-dependent cell transformation/mitogenesis at lower doses (Wells et al. 1990). From these data, it was concluded that internalization of the EGFR played a role in attenuating EGFR-mediated responses. This idea was challenged by Vieira et al. when they inhibited EGFR internalization using a dominant negative mutant of the large GTPase, dynamin. Dynamin regulates the internalization of clathrin-coated pits, and the expression of dominant negative dynamin allowed activation of the EGFR and retention at the plasma membrane. In these experiments, it was determined that full activation of MAPK and PI3-K could not be achieved if the receptor were retained on the cell surface. Conversely, the activity of some effectors (i.e., PLCγ and Shc) was enhanced by cell surface retention of the receptor. Thus, receptor internalization both positively and negatively regulates signaling (Vieira et al. 1996).

The compartmentalization of the EGFR as it moves through the endocytic pathway provides additional mechanisms to regulate receptor:effector interactions. It remains to be seen whether the signaling from a given endocytic compartment can be attributed to a specific cell physiology. If this does prove to be the case, inhibition of EGFR signaling from distinct cellular locations may be a new way to modulate receptor response.

## Ligand:Receptor Dissociation

Within 5–10 minutes of ligand stimulation, the ligand:receptor complex enters the cell and traffics to the early endosome. The early endosome, as well as all subsequent endosomes, is characterized by its increasingly acidic environment. The early endosome is recognized as a point of sorting in the cell where cargoes are directed to a variety of cellular locations such as the late endosome, endoplasmic reticulum, or to the plasma membrane. For the EGFR, it has been shown that all three routes are viable options and dependent on the cell type. However, ligand-stimulated EGFR degradation is a saturable process, and trafficking to the lysosome may be the primary destination of the ligand:receptor complex in cells with the physiologic levels of receptor and trafficking proteins (French et al. 1994; Mizuno et al. 2005). Similarly, recycling of the stimulated EGFR back to the plasma membrane may be the consequence of receptor overexpression (Masui et al. 1993). Targeting to the ER and onto the nucleus is a relatively new model, but has been shown in multiple cell lines and affects the transcription of cyclin D, and important regulator of cell cycle regulation (Liao and Carpenter, 2007).

The molecular mechanisms of some aspects of early endosomal sorting have been well-established. Binding of ligand to the EGFR is pH sensitive, with optimal binding occurring at physiological pH and dissociation occurring at lower pHs. The lower pH of the early endosome can cause dissociation of the receptor from the ligand. However, all ligand:EGFR interactions are not affected equally by pH. For instance, TGF-α is more sensitive to the early endosome pH and therefore dissociates more readily in the early endosome (Korc and Finman, 1989). Once free of ligand, the receptor becomes rapidly desphosphorylated, thereby inhibiting interactions with downstream effectors, such as c-Cbl (Lenferink et al. 1998). In the absence of c-Cbl association and receptor ubiquitylation, the receptor is not properly targeted to the lysosome for destruction and instead recycles to the plasma membrane. Thus, the TGF-α:EGFR dissociation induced in the early endosome has the immediate consequence of receptor inactivation, but the potential for multiple rounds of receptor activation and enhanced signaling.

If one considers the models of compartmentalized signaling, a strategy of ligand:receptor dissociation may be effective for inhibiting EGFR signals from the late endosome/MVB, as the activated complex would never enter that compartment. To date, it has not been shown that unique or significant signaling originates from the late endosomes, although this remains a formal possibility. Overall, ligand:receptor dissociation has limited potential as a mechanism for inhibiting EGFR signaling, unless additional measures could be taken to ensure the unbound receptor were targeted for degradation.

## Inactivation by Protein Tyrosine Phosphatases

A second mechanism by which EGFRs are inactivated is the catalyzed dephosphorylation of the receptor by phosphatases. The discovery of protein-tyrosine phosphatases (PTP) came several years after that of tyrosine kinases, but they were immediately recognized as important regulatory components of signaling (Tonks et al. 1988). Disruption of the reciprocal interactions between kinases and phosphatases can result in dramatic changes in cell physiology (Tonks and Neel, 2001). All PTPs share a central catalytic domain, while the differences in their amino and carboxy terminus confer unique cellular locations and binding partners (Barford et al. 1995). PTPs dephosphorylate substrates through the formation of a covalent bond between a phosphatase cysteine residue and the substrate phosphate followed by hydrolysis (Cirri et al. 1993; Zhang and VanEtten, 1991).

There are approximately 100 members of the PTP superfamily. Thirty-eight (38) comprise the “classical” tyrosine-only specific subfamily, and the remainder belonging to the dual specificity phosphatases (DSPs) which dephosphorylate tyrsosine, threonine or serine residues (Tonks, 2006). To date, more is known about tyrosine phosphatase regulation of EGFR signaling than regulation by dual specificity phosphatases. The tyrosine-only phosphatases can be further divided into the transmembrane receptor-like PTPs and the intracellular non-receptor PTPs (Andersen et al. 2004). Both receptor and non-receptor tyrosine phosphatases have been shown to directly regulate EGFR activity (Suarez Pestana et al. 1999; Tomic et al. 1195). This regulation can occur at multiple levels: by choice of protein substrate (receptor), recognition sequence, and subcellular localization.

The development of “substrate-trapping” mutants of phosphatases has proved to be an invaluable tool for identifying specific substrates. Through mutation of either critical residues of the active site (cysteine) or the catalytic domain (asparigine), these mutants overcome the transient nature of the enzyme-substrate complex to retain high affinity binding of phosphatases to their substrates (Flint et al. 1997). These mutatants were key in the identification of the EGFR as a substrate for numerous phosphatases. Substrate trapping mutants were expressed in COS1 cells and used to co-immunoprecipitate the T-cell protein tryosine phosphatase (TCPTP) and the EGFR. Subsequently it was shown that overexpression of transfected TCPTP, but not the substrate-trapping mutant, dephophorylated the receptor in an EGF-dependent manner, indicating the presence of a regulatory feedback mechanism (Tiganis et al. 1998). In a subsequent study in COS1 cells, TC45, the nuclear form of TCPTP, was transiently overexpressed and examined for effects on EGF-induced activation of specific signaling pathways. Overexpression of TC45 caused a decrease in EGFR phosphorylation and reduced signaling to downstream effectors, namely PI3-K- mediated activation of Akt (Tiganis et al. 1999). Similarly, in U87MG cells, a glioblastoma-astrocytoma cell line that normally expresses low levels of TC45, stable overexpression of TC45 negatively regulates the receptor and decreased both Akt and MAPK signaling (Klingler-Hoffmann et al. 2001). Together, these studies illustrate how the targeted dephosphorylation of the EGFR can decrease the activity of effectors that lead to cell growth.

The specificity of a phosphatase can also be intramolecular. The existence of site-specific phosphatases that differentially dephosphorylate particular phospho-tyrosines on the carboxy terminus of the EGFR is well established. The loss of these phosphotyrosines allows for regulation of individual EGFR signaling pathways. Using the previously described substrate-trapping mutants, the direct association between receptor-type protein-tyrosine phosphatase-κ (RPTP-κ) and the EGFR was demonstrated when the two proteins were co-expressed in Chinese Hamster Ovary cells which do not normally express either protein. The RPTP-κ has been shown *in vitro* to rapidly dephosphorylate EGFR at phosphotyrosine residues 1068 and 1173, but not phosphotyrosine 992. While overexpression of RPTP-κ in human keratinocytes led to decreased levels of phosphorylated EGFR and growth, RNAi mediated knockdown had the reciprocal effect of increasing basal and EGF-mediated phosphorylation levels and MAPK activity (Xu et al. 2005).

Tyrosine-residue specific dephosphorylation has also been identified for the non-receptor SH2-domain containing PTPs, SHP-1, and SHP-2. SHP-1 associates directly with the EGFR in human mammary carcinoma cells through phosphotyrosine 1173 on the EGFR (Keilhack et al. 1998; Tomic et al. 1995), while SHP-2 mediates the dephosphorylation of tyrosine 992 (Agazie and Hayman, 2003). Overexpression of SHP-1 in human epithelial cells led to the downregulation of MAPK activity, while expression of the tyrosine 1173 to phenylalanine mutant (Y1173F) EGFR mutant, prevents SHP-1 binding to the receptor, and leads to enhanced EGFR-mediated MAPK signaling (Keilhack et al. 1998).

The third mechanism by which phosphatase activity is regulated is by restriction of its cellular distribution, thereby controlling where in the cell EGFR signaling is terminated. By adding back a fluorescently-tagged, catalytically inactive PTPB1 mutant to PTP1B−/− mouse fibroblasts, Haj et al. were able to determine the subcellular localization at which the modified phosphatase associated with a GFP-tagged EGFR (Haj et al. 2002). As expected, the ligand-stimulated GFP-EGFR internalized. Once inside the cell, the EGFR trafficked to the ER where PTPB1 association was determined by Fluorescence Energy Transfer (FRET). The authors propose this to be the site of dephosphorylation, and suggest this occurs prior to the EGFR being targeted to the lysosome. The authors speculate that there may be “dephosphorylation compartments” at other locations within the cell. Intriguingly, they propose that endocytosis of the receptor may potentiate signaling by removing the receptor from plasma membrane localized PTPs.

It is important to note that increases in phosphatase activity are not always associated with decreased EGFR signaling. Increased phosphatase activity can also positively regulate EGFR signaling. For instance it has been shown than SHP-2 specifically dephosphorylates phosphotyrosine 992 of the EGFR (Agazie and Hayman, 2003). Interestingly, the *Drosophila* homolog of SHP-2 dephosphorylates the binding site for the SH2 domain of the Ras GTPase activating protein (RasGAP), which hydrodylzes GTP bound Ras to GDP Ras. Therefore, positive regulation of EGFR through inhibiting the activity of a negative regulator of downstream EGFR signaling seems the most likely mechanism of SHP-2′s positive effect on EGFR signaling activation.

When considering strategies to attenuate EGFR signaling, there are a number of options including enhanced phosphatase recruitment, expression, and activation of receptor specific inactivating phosphatases and inhibition of phosphatases, such as SHP-2, that positively regulate signaling. However, prior to phosphatase becoming a therapeutic target, several important questions regarding molecular mechanism must be answered. For instance, is the phosphatase specific for the EGFR or will other receptors also be affected? Are the tyrosines that are dephosphorylated going to affect signaling pathways required for cell growth and/or survival? Are the changes in phosphatase activity going to positively or negative regulate signaling? Nevertheless, the fact the modulation of phosphatase activity and expression in tissue culture models causes measurable changes in cell physiology, strongly supports the idea that phosphatases are viable therapeutic targets.

## Trafficking Mediated EGFR Inactivation

As mentioned previously, the EGFR remains active during its endocytic trafficking. Both the phosphorylated receptor and downstream effectors have been isolated from early endosomes (DiGuglielmo et al. 1994; Lai et al. 1989). There are two logical points in the late endocytic pathway in which receptor inactivation may occur. The first point is when the ligand receptor complex gets sequestered into the intralumenal vesicles of the MVB. This causes the physical separation of the receptor and downstream effectors. However, it is controversial as to whether this event functionally attenuates receptor-mediated signaling. The second potential point of signal termination is the lysosomal degradation of the receptor. While this will clearly terminate the signaling process, since it follows receptor sequestration it may not be physiologically relevant.

### EGFR sequestration

The appearance of the ligand-stimulated EGFR in multivesicular endosomes has been reported by numerous groups in a variety of cell types (Carpentier et al. 1987; Dunn et al. 1986; Miller et al. 1986). The ultrastructural analysis of liver carcinoma cell lines revealed stimulation with EGF stimulates MVB biogenesis and increases the number of internal vesicles in MVBs (White et al. 2006). The data indicating the liganded EGFR facilitates receptor sequestration into MVBs allows for the generation of a model in which this process separates the receptor and effector, thereby limiting the duration of receptor signaling. Until recently, there were no data that indicated that the removal of the activated receptor from the cytosol to the intralumenal vesicle affected signaling. Thus, it was unclear whether entry into MVB was a regulatory mechanism for EGFR signaling or an intermediate step on the path to lysosomal degradation.

EGFRs destined for recycling are retained on the limiting membrane of the early endosome. Receptors that make it to the late endosome/MVB accumulate onto areas of the limiting membrane of the late endosome that invaginate and pinch off to form internal vesicles within the late endosome/MVB. Sequestration of the EGFR within MVBs not simply a passive event, as receptor activation mediates this lysosomal sorting through a series of highly conserved, regulated, and concerted steps (Felder et al. 1990). The molecular identity of many of the proteins involved in this sorting were first identified in yeast genetic studies and are referred to as the vacuolar protein sorting (Vps) proteins (Katzmann et al. 2002; Raymond et al. 1992). The role of an increasing number of mammalian homologs has been confirmed in tissue culture models (Babst, 2005).

Targeting of the EGFR for degradation begins with the phosphorylated receptor associating with the E3 ubiquitin ligase c-Cbl, ubiquitylation of the receptor, and targeting of the receptor for degradation. Ubiquitylation of the EGFR has an established role in sorting the receptor for lysosomal degradation, but the role of this process in internalization remains controversial (Duan et al. 2003; Huang et al. 2006; Jiang and Sorkin, 2003). The current model for sorting is recognition of the ubiquitinated receptor by the ubiquitin-interacting motif (UIM) of hepatocyte growth factor-regulated tyrosine kinase substrate (Hrs) in complex with STAM-1 (Bache et al. 2003; Urbe et al. 2003). Together, these proteins recruit related protein complexes, known as the ESCRT (Endosomal Sorting Complex Required for Transport) complexes I, II, and III (Hurley and Emr, 2006). These complexes, along with several other highly conserved proteins, are responsible for directing the ligand: receptor complex to the intralumenal vesicles and subsequent lysosomal degradation (Table 1).

### ESCRT mediated EGFR sequestration

Through mediation of MVB biogenesis and lysosomal sorting, proteins implicated in the late endocytic trafficking of the EGFR can impact signaling. Multiple lines of evidence, in both mammalian and *C. elegans* models, indicate the Cbl-mediated ubiquitination of the EGFR negatively regulates EGFR signaling (Levkowitz et al. 1999; Yoon et al. 1995). The *hrs* deletion mutants in *Drosophila* reveal impairment in MVB formation leading to sustained EGFR phosphorylation and MAPK activity. The phenotype of *hrs* deletion mutants is the embryonic expansion of cells dependent on the EGFR during development. Therefore, the role of the earliest mediators of lysosomal sorting in signaling is well documented, whereas the role of the remaining mediators is only beginning to be understood.

Recent studies by Bache et al. examined the contribution of proteins from the ESCRT-I or ESCRT-III complex in EGFR trafficking and signaling. Using HeLa cells as a model, two ESCRT proteins, Tsg101 (ESCRT-I), and Vps24 (ESCRT-III) were independently knocked down by RNAi (Bache et al. 2006b). While the knockout of either protein caused significant delays in ligand-stimulated receptor degradation, only knockdown of Tsg101 sustained EGFR signaling to the effectors MEK and MAPK. Using high-resolution electron microscopy, the authors observed that knockdown of Vps24 caused the accumulation of the EGFR within internal vesicles of endosomes that appeared smaller than typical MVBs. A failure to sequester the EGFR and retain it on the limiting membrane of MVBs has been previously reported when TSG101 levels are depleted by RNAi (Razi and Futter, 2006). These results support the model that sorting to lumenal vesicles of late endocytic compartments sufficiently terminates receptor signaling. A link between Tsg101 function and the uncontrolled growth characteristic of cancer cells is suggested by a study in which Tsg101 was inactivated by mRNA antisense transcripts, which led to increased colony formation in soft agar. Further the Tsg101 antisense transcripts, when injected into nude mice, increased the appearance of metastatic tumors (Li and Cohen, 1996).

While the work by Bache et al. provide a compelling argument that receptor sequestration is a key regulatory mechanism terminating EGFR signaling, there are data that contradict this model. Other studies suggest that signaling can be terminated despite defects in intralumenal vesicle sequestration (Malerod et al. 2007). Depletion of Vps22 by siRNA in HeLa and HEp2 cells causes a slowed EGFR degradation and accumulation of the receptor along the limiting membranes of the MVB as shown by electron microscopy and biochemical analysis. However, whereas this study, in accordance with previous reports, showed TSG101 knockdown enhances EGF-dependent MAPK phosphorylation, Vps22 knockdown did not prolong MAPK activity as compared to wild type cells. Interestingly, the authors also found that lysates from Vps22 siRNA treated cells have reduced levels of two other ESCRT-II proteins, Vps25 and Vps36. Therefore, Vps22 may contribute to the stability of the entire complex. The signaling data in Vps22 depleted cells highlight the controversy regarding whether receptor inactivation can occur upstream of intralumenal sequestration of the EGFR and the exact molecular role of each of the ESCRT complexes.

### Non-ESCRT medicated EGFR sequestration

Proteins that are not part of the ESCRT complex also have been shown to play a role in EGFR intralumenal sequestration. For instance, the EGFR effector annexin-1 has been shown to be required for sequestration (Futter et al. 1993). Despite the failure of the EGFR to internalize into vesicles within the MVB in annexin-1 mouse knockout cells, the EGFR is still efficiently degraded, albeit with a minor delay in the rate of degradation (White et al. 2006). The authors suggest that internal vesicle formation is not necessary for EGFR degradation, but makes the degradation process more efficient. Thus, entry of receptors into limiting membranes of the MVB may in fact be to sequester the receptor rather than simply as an intermediate in the degradation pathway. Similar results have been seen with the knockdown of Vps34, a PI-3 kinase, in HEp-2 cells (Futter et al. 2001). Like the annexin-1 knockout, the active EGFR is retained on the limiting membrane of the late endocytic compartment and there is only a slight delay in the rate of delivery to lysosomes. Examination of the tyrosine-phosphorylation of downstream effectors in Vps34 knockdown cells revealed enhanced phosphorylation of several proteins. One implication of these findings is that there are two possible points of inactivation in the late endocytic pathway since, in the absence of sequestration-mediated inactivation, lysosomal delivery functions as the mechanism of receptor inactivation.

### EGFR degradation

There is evidence that sequestration of the EGFR is not sufficient for terminating the signaling capability of the EGFR and lysosomal degradation is the rate-limiting step in receptor inactivation. Oksvold et al. prevented lysosomal degradation using inhibitors of lysosomal enzymes or blocked trafficking to lysosome using the lysosomotropic amine chloroquine (Oksvold et al. 2001). Immunoblots of subcellular fractions isolated over gradients revealed the accumulation of activated EGFR and MAPK within the same fraction. Immunoelectron microspcopy revealed the majority of phosphorylated EGFR accumulated intralumenally, with a small fraction present on the cytosolic membrane. Immunofluorescent staining of cells treated with chloroquine or lysosomal inhibitors showed co-localization of the adaptor proteins Shc, Grb2, and Cbl with phosphorylated EGFR indicating the potential for signaling existed. These proteins accumulated in MVB as defined by the presence of MVB markers CD63 and LAMP-1, and exclusion of the early endosome marker EEA1. Immunoblot analysis of phosphorylated EGFR and MAPK revealed cells stimulated with EGF and chased with chloroquine for 120′, but not untreated cells, sustained their activity at time points that electron microscopy revealed to be consistent with EGFR localization in MVBs. These data indicate that in the absence of lysosomal delivery, activated receptor can accumulate in MVBs and signal to downstream effectors.

In the pharmacologically treated cells, immunoelectron microscopy revealed the majority of phosphorylated receptors accumulated in internal vesicles of the MVB. It is a formal possibility that sustained signaling was due to the fraction remaining on the limiting membrane. EGF stimulation substantially increases the volume of cytosol containing MVBs (Futter et al. 1998; White et al. 2006); suggesting proteins other than the EGFR, such as downstream effectors, could be removed from the cytosol and delivered for degradation. Therefore, the presence of an activated effector is not definitive evidence that physiologic signaling is occurring. Further analysis of downstream signaling events, such as DNA transcription, cell viability, and cell motility is needed to establish whether the active effectors modulate cell biology.

The molecular regulation of EGFR signaling in the late endocytic pathway remains unclear. Specifically, determining whether the terminal step in EGFR signaling is mediated through receptor sequestration or degradation will aid in the design and use of therapeutic agents. To date, the majority of studies have focused on disrupting the sequestration/degradation process. As a strategy to terminate hyperactive EGFR signaling, it will be more useful to accelerate these trafficking events and examine the biochemical (effector activity) and physiological (cell biology) consequences. These findings will determine the potential of the late endocytic pathway as a therapeutic target.

## Concluding Remarks

Clinical and experimental data indicate that the EGFR is a viable target to inhibit the growth of non-transformed and transformed cells. Monoclonal antibodies targeting the EGFR and tyrosine kinase inhibitors have both demonstrated success in inhibiting the growth of cancerous and non-cancerous cells. However, there still remain a number of cancers characterized by EGFR overexpression that are refractory to these therapies. Rather than strive to make better inhibitors of EGFR activity, we suggest the alternative approach of looking for ways to accelerate the inactivation of the EGFR once it becomes stimulated. This strategy would have the greatest effect on those cells with the highest levels of EGFR expression and EGFR activation. As the biochemical details of the pathways that lead to receptor inactivation are elucidated, it allows one to creatively think about how this information can be used in anti-cancer-therapies.

## Figures and Tables

**Table 1:** Role of various proteins in EGFR trafficking and signaling.

Complex/Protein	MVB	EGFR degradation with KD	EGFR signaling with KD	Citation
**ESCRT- I**				
Tsg101/Vps23	Inhibits MVB biogenesis	↓ ↓ ↓	Sustained MAPK activation	(Babst et al. 2000; Bache et al. 2006b; Doyette et al. 2005; Malerod et al. 2007; Olabisi et al. 2006; Razi and Futter, 2006)
VPS28/Vps28	N.D.	Inhibits degradation (antibody)	N.D.	(Bishop et al. 2002)
VPS37/Vps37	N.D.	↓ ↓	N.D.	(Bache et al. 2006a)
**ESCRT-II**				
EAP30/Vps22	Decrease in EGFR ILV sequestrastion	↓ ↓	MAPK—no change	(Malerod et al. 2007)
EAP20/Vps25	N.D.	No change (Bowers) ↓ ↓ (Langelier et al.)	N.D.	(Bowers et al. 2006; Langelier et al. 2006)
**ESCRT-III**				
CHMP6/Vps20	N.D.	↓	N.D.	(Langelier et al. 2006)
CHMP3/Vps24	Decreased size of MVBs	↓	MAPK—no change	(Bache et al. 2006b; Yan et al. 2005)
**Other**				
Hrs/Vps27	Increased MVB size; decreased ILVs	↓	↑ MAPK	(Bache et al. 2003; Lu et al. 2003; Malerod et al. 2007; Razi and Futter, 2006)
Rab7	N.D.	↓ ↓ (dominant neg) ↓ ↓ ↓ (knockdown)		(Ceresa and Bahr, 2006) personal communication
Vps4/Vps4	Reduced # of ILVs (mutant)		N.D.	(Sachse et al. 2004)
Vps34	Decreased ILV formation	↓	N.D.	(Futter et al. 2001)
UBPY/Doa4	Increased # and size of MVB; fewer ILVs	↓ ↓	N.D.	(Row et al. 2006)
LIP5/Vtal	N.D.	↓ ↓ ↓	N.D.	(Ward et al. 2005)

**Notes:** Shown in a partial listing of proteins that have been shown to have a role in EGFR trafficking through the late endocytic pathway. N.D. = not determined. Downward arrows represent the change in EGFR degradation kinetics. ↓ ↓ ↓, ↓ ↓, and ↓ indicate an increase in the half-life of ligand-stimulated EGFR by >5-fold, 3–4 fold, and 1–2 fold, respectively. Unless otherwise noted, studies were done by knocking down protein expression. # refers to the number. ILV = Intralumenal vesicles.
